# Presence of Antibiotic Residues and Antibiotic Resistant Bacteria in Cattle Manure Intended for Fertilization of Agricultural Fields: A One Health Perspective

**DOI:** 10.3390/antibiotics10040410

**Published:** 2021-04-09

**Authors:** Judith Huygens, Els Daeseleire, Jacques Mahillon, Daan Van Elst, Johan Decrop, Jurgen Meirlaen, Jeroen Dewulf, Marc Heyndrickx, Geertrui Rasschaert

**Affiliations:** 1 Technology and Food Science Unit, Flanders Research Institute for Agriculture, Fisheries and Food (ILVO), Brusselsesteenweg 370, 9090 Melle, Belgium; els.daeseleire@ilvo.vlaanderen.be (E.D.); daan_van_elst@hotmail.com (D.V.E.); marc.heyndrickx@ilvo.vlaanderen.be (M.H.); geertrui.rasschaert@ilvo.vlaanderen.be (G.R.); 2 Laboratory of Food and Environmental Microbiology, Earth and Life Institute, UCLouvain, 1348 Louvain-la-Neuve, Belgium; jacques.mahillon@uclouvain.be; 3 Flemish Land Agency (VLM), Manure Bank, Enforcement Nitrate Directive, 1210 Brussel, Belgium; johan.decrop@vlm.be; 4 Department of Water Reporting, Flemish Environmental Agency (VMM), 9300 Aalst, Belgium; j.meirlaen@vmm.be; 5 Department of Reproduction, Obstetrics and Herd Health, Faculty of Veterinary Medicine, Ghent University, Salisburylaan 133, 9820 Merelbeke, Belgium; jeroen.dewulf@ugent.be; 6 Department of Pathology, Bacteriology and Avian Diseases, Faculty of Veterinary Medicine, Ghent University, Salisburylaan 133, 9820 Merelbeke, Belgium

**Keywords:** antibiotic residues, resistance, cattle manure, *E. coli*, *Salmonella*

## Abstract

Antibiotic resistant bacteria and antibiotic residues can enter the environment when using animal manure as fertilizer. Twenty-five mixed beef cattle farmyard manure samples and 9 mixed fattening calf slurry samples from different farms across Belgium were investigated for the presence of 69 antibiotic residues, antibiotic resistant *Escherichia coli* and *Salmonella* spp. Doxycycline, oxytetracycline, ciprofloxacin, enrofloxacin, flumequine and lincomycin were detected in all fattening calf slurry samples with mean concentrations of 2776, 4078, 48, 31, 536 and 36 µg/kg manure, respectively. Sulfadiazine was detected at a mean concentration of 10,895 µg/kg. Further, antibiotic residues were found in only 4 of the 25 beef cattle farmyard manure samples. Oxytetracycline was detected twice below 500 µg/kg. Paromomycin, ciprofloxacin and enrofloxacin were detected in a concentration below 100 µg/kg. Of *E. coli* isolates, 88% and 23% from fattening calf slurry and beef cattle farmyard manure, respectively, were resistant to at least one of the antibiotics tested. Multi-drug resistance was observed at a maximum of 10 and 7 antibiotics, respectively. The occurrence of antibiotic resistant *E. coli* and antibiotic residues is shown to be higher in fattening calf slurry than in beef cattle farmyard manure used for agricultural field fertilization.

## 1. Introduction

Antibiotic resistance is a worldwide threat to human and animal health. According to a joint FAO-OIE-WHO report, antibiotic resistance is among the top three priorities of health risks within the area of zoonotic diseases [1]. The worldwide intensive use of antibiotics in human and veterinary medicine has accelerated the selection of antibiotic resistant bacteria [2]. Therapy failure of bacterial infections in humans and animals is one of the most important consequences of antibiotic resistant bacteria [3]. Antibiotic resistance is responsible every year for 700,000 deaths worldwide, with up to 10 million deaths predicted per year in 2050 if nothing happens to reduce the increase of resistance development [4]. Despite the projected increases in antibiotic resistance the worldwide consumption of antibiotic agents remains high. The region of Asia-Far East-Oceania is estimated to have the highest use of antibiotic agents (expressed per kg animal) followed by the Americas, Europe, and Africa, respectively [5]. In 2018, within 31 European countries 6431.4 tons of active substance of antibiotics were sold for food-producing animals, corresponding to an average of 103.2 mg veterinary antibiotic agents sold per PCU (population correction unit). The PCU was used as a proxy for the biomass represented by the food-producing animal population [6]. Particularly in Belgium, high livestock density and intensive arable farming are coupled with high antibiotic use. In Belgium in 2018, 113.1 mg/PCU veterinary antibiotic agents were sold, which is above the European mean [6]. Animals are exposed to antibiotics by group treatments and individual treatments [7]. The resulting selection pressure can cause colonization of antibiotic resistant bacteria in the microbiota of the gut [8].

Furthermore it is considered that 30–90% of the antibiotics administered for veterinary use can be excreted in manure or urine, either unchanged or as an active metabolite [9,10]. Raw manure applied on the agricultural field is one of the ways that antibiotic residues and antibiotic resistant bacteria can enter the environment. Through this process, manure and soil associated bacteria can exchange antibiotic resistance genes by horizontal gene transfer [11]. Those antibiotic residues and resistant bacteria in soil may end up in surface water, may leach into groundwater, and may be taken up by crops growing on fertilized fields, thus possibly posing a risk for human and animal exposure through consumption of food and feed [3,8]. Those transmission routes seem to be relevant as the overall manure production in northern Belgium (Flanders) in 2019 was 127 kilotons of nitrogen (N), 72% of which were used as fertilizer [12]. Specifically, cattle manure is one of the main sources of N (70 kilotons), 94% of which was applied on agricultural fields [12]. In southern Belgium (Wallonia region), 91.2% of the total manure production each year comes from cattle (54 kiloton N), 71% of which is cattle farmyard manure. [13].

Although antibiotic resistance is an urgent problem with a large impact on public health, little is known about the presence of antibiotic agents and antibiotic resistant bacteria in animal manure. Even though the veal sector is not the largest animal production sector in Belgium, the antibiotic use (expressed in BD_100_-species, defined by the treatment days out of 100 days based on the total amount of antibiotics used per species and the total mass animals at risk per species) is high within the veal sector. The average BD_100_ of veal calves (22.27) was far higher than the BD_100_ of pigs (6.72) [14]. Similarly, the incidence of antimicrobial group treatment was shown to be strikingly higher in white veal calves as compared to conventional cattle and dairy cattle in Belgium [7]. The aim of this study was therefore to semi-quantify 69 antibiotic residues and determine antibiotic resistance of *Escherichia coli* in different cattle manure types intended for fertilization of agricultural fields in Belgium as this could contribute to antibiotic resistance in the environment. To do so, fattening calf slurry samples as well as beef cattle farmyard manure (FYM) samples were investigated.

## 2. Results

### 2.1. Microbiological Analyses

No *Salmonella* was detected in any of the samples. *E. coli* was detected in 8 of the 9 fattening calf slurry samples and in 22 of the 25 beef cattle FYM samples. For each sample, at least 4 isolates were investigated further, except for 3 samples in which only one *E. coli* isolate was investigated. In total, 123 *E. coli* (41 from the fattening calf slurry samples and 82 from the beef cattle FYM samples) were recovered from plates without antibiotics. A wide variety was observed in the antibiotic resistance profiles of the isolates coming from the same sample. Of these *E. coli* isolates, 88% of the isolates from fattening calf slurry and 23% of the isolates from beef cattle FYM were resistant to at least one of the antibiotics tested (Table 1). A minimum of 70% of the isolates from fattening calf slurry samples were resistant to ampicillin, sulfamethoxazole, trimethoprim and tetracycline (Table 2), which was remarkably higher than the resistance (less than 20%) observed in the beef cattle FYM isolates. Five *E. coli* isolates from fattening calf slurry samples were resistant to colistin (Table 2). A multi-drug resistance (MDR) up to 10 antibiotics was observed for the fattening calf slurry isolates versus up to 7 for the isolates from beef cattle FYM (Table 1).

Ciprofloxacin-resistant *E. coli* were detected in 7 fattening calf slurry and in 7 beef cattle FYM samples, isolated from ciprofloxacin-supplemented agar plates. Cefotaxime-resistant *E. coli* were detected in 7 fattening calf slurry and in 5 beef cattle FYM samples, isolated from cefotaxime-supplemented agar plates. A MDR was observed in 13 of the 14 ciprofloxacin-resistant *E. coli* isolates and in all 11 cefotaxime-resistant *E. coli* isolates (Tables S1–S3). No meropenem or colistin-resistant *E. coli* was isolated from the respective supplemented agar plates.

### 2.2. UHPLC-MS/MS

In fattening calf slurry, between 8 and 17 antibiotic residues were detected in each sample. The six following antibiotics were found in all fattening calf slurry samples: doxycycline, oxytetracycline, ciprofloxacin, enrofloxacin, flumequine and lincomycin. Doxycycline and oxytetracycline were found in high concentrations, namely mean concentrations of 2776 µg/kg and 4078 µg/kg, respectively, while the maximum concentrations were 10,881 µg/kg and 19,522 µg/kg, respectively. Flumequine was found in a maximum concentration of 4494 µg/kg in one sample. The median concentration was 21 µg/kg, because in the other samples only low concentrations were found. Sulfadiazine was detected in 8 of the 9 fattening calf slurry samples and was found in a mean concentration of 10,895 µg/kg with a high maximum concentration of 84,084 µg/kg. Neomycin was found in a mean (minimum-maximum) concentration of 1863 µg/kg (960–3186 µg/kg) and detected in 3 of the 9 samples. Tilmicosin was detected in 8 fattening calf slurry samples with a mean concentration of 162 µg/kg and a maximum concentration of 1149 µg/kg. Tylosin was detected in only 2 samples with a maximum concentration of 504 µg/kg. Enrofloxacin (and its metabolite ciprofloxacin), lincomycin, marbofloxacin, tetracycline and sulfadoxine were all found in mean concentrations less than 100 µg/kg and had no maximum concentrations higher than 250 µg/kg (Table 3). For all the detected antibiotic residues, a wide variation in concentrations was observed (Table 3).

Colistin A and B were detected in only one fattening calf slurry sample in a concentration of 153 µg/kg and 88 µg/kg, respectively. As the sum of colistin A and colistin B represents more than 85% of colistin by weight, a concentration around 284 µg/kg can be expected in the sample [15,16].

Only 4 of the 25 beef cattle FYM samples tested positive for antibiotic residues. Oxytetracycline was detected in 2 samples in a concentration of 28 µg/kg and 471 µg/kg, respectively. Enrofloxacin and its metabolite ciprofloxacin were detected in the same sample in a concentration of 80 µg/kg and 35 µg/kg, respectively. Paromomycin was detected in another sample in a concentration of 50 µg/kg (Table 3).

## 3. Discussion

The antibiotic resistance in the general *E. coli* population (as picked from agar plates without antibiotics added) of fattening calf slurry was higher than of beef cattle FYM (Table 1). In a Dutch study in 2018 manure samples from livestock animals were also investigated for the presence of antibiotic resistant *E. coli*. The highest resistance levels were found in white veal calves and broilers followed by slaughter pigs. Low levels of antibiotic resistance were reported in older calves and dairy cattle. Those resistance levels are in accordance with the antibiotic use in these livestock species [17]. Comparable to our isolates, the highest resistance rates were found for the most frequently used antibiotic classes: penicillins, tetracyclines and sulfonamides [6,14,17]. Antibiotic resistance in *E. coli* from fattening calves in our study, were generally higher than reported in the Dutch study for the white veal calves [17]. Antibiotic resistance in *E. coli* from our beef cattle FYM were comparable to the older veal calves and higher than dairy cattle [17]. However, it should be noted that the number of fattening calf slurry samples was rather limited in our study. Further the sampling method can have an impact on the results. The systematically higher resistance rates in our study is possibly due to the enrichment of resistant bacteria during storage in the manure pit under continuous antibiotic pressure. The proportion of samples containing cefotaxime-resistant *E. coli* and ciprofloxacin-resistant *E. coli* was also higher in fattening calf slurry. The results in this study and the comparison to previous studies are consistent with the more intensive use of antibiotics within the veal calf sector compared to the use within other animal categories. For example, in a study of pardon et al. (2012) the incidence of group antimicrobial treatment was 414.0 ADD (animal defined daily dose) per 1000 veal calves compared to 5.4 ADD per 1000 beef cattle and 235.7 per 1000 pigs. ADD is the average maintenance dose for the main indication in a well-defined animal species [7]. It is important to study the antibiotic resistance of *E. coli* that enter the environment through fertilization. Besides being an fecal indicator organism, it is for both humans and animals a commensal bacterium that can represent a reservoir of antibiotic resistant bacteria in the gut. The results of this study provides insight into the antibiotic resistance profiles of intestinal bacteria from production animals like cattle as well as their potential transfer to the human gut through the consumption of food such as vegetables fertilized with cattle manure.

Besides the indicator organism *E. coli*, cattle manure can also be a source of zoonoses such as *Salmonella* [18]. During fertilization, the risk exists that those abovementioned zoonotic bacteria come in contact with livestock or crops intended for human consumption [19]. No *Salmonella* was detected in the fattening calf slurry and beef cattle FYM samples investigated here. In contrast, in previous studies a low prevalence of *Salmonella* was reported in bovine manure, ranging from 0.2% to 10.0% [20,21,22,23]. The prevalence of *Salmonella* is also dependent on the age of cattle manure. *Salmonella* can decline by 90% or more after a manure storage of 2 to 4 weeks [24]. Furthermore, composting of bovine manure causes *Salmonella* reductions of 3–4 logs to undetectable levels [25]. However, it is striking that in pig manure 57% of the manure samples contained *Salmonella* [26]. This means that cattle manure could be a “safer” fertilizer in terms of *Salmonella* presence.

Tetracyclines were detected in relatively high concentrations. In Europe in 2018, this was the most sold antibiotic class for food-producing animals [6]. It has been reported that tetracyclines are very persistent in both solid and liquid manure from cattle [27,28], which is in agreement with our findings. During fertilization of the agricultural field, approximately a 20 to 80-fold dilution of calf slurry is made when mixing with the upper layers of the soil (personal communication, Johan Decrop from the Flemish Manure Bank VLM). Assuming that range of dilution, within the present study doxycycline and oxytetracycline would be found in estimated concentrations of 6–544 µg/kg and 1–976 µg/kg in the soil, respectively. Gullberg et al. [29] have described a selection of antibiotic resistant bacteria at an in vitro concentration of 15 µg/L tetracycline. Moreover, Thomaidi et al. (2016) calculated predicted no-effect concentrations for antibiotic resistance selection in soil (PNEC_soil_). Bengtsson-Palme et al. (2016) calculated this for water (PNEC_water_). If PNEC_soil_ values were not found in the publication of Thomaidi et al. (2016), we have calculated them by multiplying the PNEC_water_ by the K_d_, which is the soil-water partition coefficient (L/kg) [30,31]. However, the PNEC_soil_ is highly dependent on both the antibiotic residue and the soil type (e.g., clay vs. sand) so the Kd value may have a broad value range.

According to the literature, the K_d_ of oxytetracycline and tetracycline ranges from 417 to 1026 L/kg [32]. Using these values in the present study tetracyclines were found in manure at concentrations above the estimated PNEC_soil_ (between ca. 200 µg/kg and 500 µg/kg) and could consequently result in resistance selection in the environment.

Notice that our concentrations are semi-quantitative as the quantification of antibiotic residues in fattening calf slurry was done by one-point standard addition. It is sufficient to study the occurrence of antibiotic residues in a certain concentration range instead of a precise quantification. Although the results should be considered as semi-quantitative, we compared them with modelled concentrations or found in literature, considered to have an impact on resistance selection.

Sulfadiazine was frequently detected in the fattening calf slurry samples in varying concentrations. The sulfonamides were the third most sold antibiotic class in food-producing animals in Europe in 2018 [6]. Taking the 20 to 80-fold dilution of fattening calf slurry in soil into account, sulfadiazine concentrations between 0.05 µg/kg and 4204 µg/kg with 0.06–0.25 µg/kg as median concentration can be expected in the soil. A PNEC_soil_ for resistance selection of sulfamethoxazole is calculated between 22 µg/kg and 224 µg/kg [31,32]. Those values and our data indicate that resistance selection for sulfonamides in the environment can occur but will be rather rare.

Enrofloxacin and its metabolite ciprofloxacin were detected in every fattening calf slurry sample, which is comparable to other studies [33]. In 2018, 2.5% of the sold antibiotics in Europe for food-producing animals were fluoroquinolones [6]. According to the World Health Organization, fluoroquinolones are listed among the most critically important antimicrobials [34]. In Belgium the poultry sector has the largest use of fluoroquinolones, followed by the veal calves [14]. As enrofloxacin was still frequently detected in the fattening calf slurry samples, the use of enrofloxacin in the veterinary sector must be reduced further to avoid the further spread of resistance. This is emphasized by the fact that resistance selection can potentially occur in the environment at the recovered concentrations in manure. More specifically, the estimated PNEC_soil_ for resistance selection of enrofloxacin are between 0.03 µg/kg and 359 µg/kg [31,32], while enrofloxacin concentrations in soil up to 8 µg/kg can be expected.

In the beef cattle FYM, oxytetracycline, ciprofloxacin, enrofloxacin and paromomycin were detected in only a few samples (Table 3). As the samples may contain manure with an age up to 15 months, the possibility exists that antibiotic residues had already degraded during storage or composting [33]. Youngquist et al. showed that nearly all 16 antibiotics in the study (which included sulfonamides, tetracyclines, quinolones, macrolides and one amphenicol) were reduced in livestock manure after composting at several conditions, with half-lives between 0.9 and 16 days [35]. Despite the low detection, the risk still exists that antibiotic residues in beef cattle FYM enter the environment because the manure is stored in piles on the agricultural field before fertilization, thus possibly resulting in hotspots of antibiotic leakage into the soil. Further it cannot be excluded that the relatively high LODs may explain the very low detection frequency of aminoglycosides. For example, paromomycin was detected in a concentration of 50 µg/kg while the mean LOD of the aminoglycosides using UHPLC-MS/MS were between 40 µg/kg and 210 µg/kg for beef cattle FYM (Table 4).

In literature, the most reported antibiotics in animal manure are tetracyclines, fluoroquinolones and sulphonamides [33]. In our study we found that lincomycin and flumequine are also important components, as they were detected in each fattening calf slurry sample and previously reported as being very persistent in animal manure. Together with enrofloxacin, they can be found in animal manure after one year at more than 10% of their original concentration [27]. The persistence of antibiotics in manure is an important characteristic for the development of antibiotic resistance in the overall environment, as manure can have long storage times before application on the field. Since manure is applied on the agricultural field, antibiotic resistant bacteria and persistent antibiotic residues enter the environment. Through this route antibiotic residues and antibiotic resistant bacteria can spread further to crops. In recent years several studies have reported the presence of antibiotic residues and antibiotic resistance in vegetables as a result of fertilization [36,37,38]. They demonstrated that different antibiotic residues and antibiotic resistant bacteria were detected in plants fertilized with raw manure. Yet more research is still needed to understand the behavior and persistence of the different antibiotic residues and the bacterial resistance in the environment in order to estimate the exposure to human and animals by the consumption of vegetables and feed.

## 4. Conclusions

To summarize, the beef cattle sector uses fewer antibiotics in comparison to the veal production sector. It is assumed that the higher antibiotic use in the fattening or veal calf sector explains the higher concentrations and higher detection rates of antibiotic residues in fattening calf slurry and the higher antibiotic resistance rate in *E. coli* isolated from fattening calf slurry. This results in a higher risk of the spread of antibiotic resistance in the environment when applying this slurry as fertilization on agricultural fields.

## 5. Materials and Methods

### 5.1. Sampling

Nine fattening calf slurry samples and 25 beef cattle FYM samples were collected in Belgium, all from different farms. The farms were selected in part based on the location of the corresponding sector. The veal calf sector is concentrated in Flanders (the northern region of Belgium), while in Wallonia (the southern region of Belgium) bovine cattle are responsible for the highest manure production [7,13]. The fattening calf slurry samples were taken from March to April 2017 when the slurry was being applied to agricultural fields. This slurry consisted of a mixture of old (maximum 6 months) and fresh manure. The beef cattle FYM samples were taken from December 2017 to March 2018 at the time the manure was piled on the agricultural field for further composting before being incorporated into the soil. The farmyard manure was a mixture of old (maximum 15 months) and fresh manure composed of manure, urine and straw. Ten manure samples were collected from suckler cows, 2 manure samples from fattening young bulls, 1 manure sample from fattening heifers, 2 manure samples from young cows speculated for milk production on later age, 1 manure sample from fattening cows, and 9 manure samples from unspecified categories. The samples were transported to the laboratory under cooled circumstances, stored at 4 °C and processed within 3 days. The fattening calf slurry samples were collected by the Flemish Land Agency (VLM, Manure Bank) and were homogenized according to BAM/part3/01 [39]. The beef cattle FYM samples were collected by Centre de Michamps (Michamps, Belgium) and homogenized according to a validated method [40,41,42].

### 5.2. Microbiological Analysis

Classical bacteriological analyses were performed to detect and identify resistant *Salmonella* and resistant *E. coli* strains. From each manure sample 25 g was mixed with 225 mL buffered peptone water (BPW, Oxoid, Basingstoke, UK). After homogenization, the mother suspension was diluted in Ringer diluent (Oxoid, Basingstoke, UK) until 10^−2^. From each dilution 100 µL was pipetted and spread using glass beads on RAPID’ *E. coli* 2 agar (Bio-Rad, Temse, Belgium) plates. This medium distinguishes *E. coli* from other coliforms. After incubation at 44 °C during 24 h, at least 4 purple colonies (*E. coli*) per sample were further purified. In that way *E. coli* without a preselection for resistance were investigated as an estimation of the antibiotic resistance in the general *E. coli* population [43]. In addition, to estimate the level of *E. coli* resistance to several antibiotics considered as critically important within human medicine, 100 µL of the mother suspension was also plated onto plates containing one of the following antibiotics; meropenem, colistin, cefotaxime or ciprofloxacin (all Sigma-Aldrich, Diegem, Belgium) at concentrations corresponding to their epidemiological cut-off values (ECOFF) for *E. coli,* which were 0.125 mg/L, 2 mg/L, 0.025 mg/L and 0.064 mg/L, respectively [44]. After incubation, from each sample one isolate grown on plates containing one of the 4 antibiotics was further purified. All collected isolates were stored at −80 °C until antimicrobial susceptibility testing was performed using Sensititre^TM^.

For isolation of *Salmonella*, the mother suspension was incubated at 37 °C for 18 h. In total, 100 µL of the enriched suspension was inoculated on Modified Semi-solid Rappaport Vassiliadis agar (MSRV, Oxoid, Basingstoke, UK) followed by incubation at 41 °C for 48 h. Suspected zones were transferred to Xylose Lysine Deoxycholate agar (XLD, Oxoid, Basingstoke, UK) and incubated at 37 °C for 24 h for isolation.

The isolates were further analyzed and interpreted using a Sensititre^TM^ microbroth dilution analysis with EUVSEC plates (TREK Diagnostic Systems, West Sussex, UK) as reported by Rasschaert et al. [26]. The MICs of the following panel of antibiotic agents were determined to set up an antimicrobial resistance profile: ampicillin, cefotaxime, ceftazidime, meropenem, nalidixic acid, ciprofloxacin, tetracycline, colistin, gentamicin, trimethoprim, sulfamethoxazole, chloramphenicol, azithromycin and tigecycline.

### 5.3. Extraction Procedure and UHPLC-MS/MS Analysis

Each manure sample was screened for 69 antibiotic residues using UHPLC-MS/MS analysis (Table 5) following 1 transition per compound. In case a signal was obtained, a confirmation step was done by re-injecting the sample where minimum 2 transitions were followed. The precursor ion, product ion and retention time of all 69 antibiotic residues is documented in Rasschaert et al. (2020) [26]. The selection of the antibiotic residues is based on an accredited screening method for the detection of antibiotic residues in milk and meat in the context of food safety for which maximum residue limits (MRL) are described in the regulation (EU) nr. 37/2010. This method includes as good as all antibiotics registered for food-producing animals in Belgium [45].

For the fattening calf slurry samples, the same extraction procedures and UHPLC-MS/MS methods were used as described in Rasschaert et al. (2020) [26]. For the multi-residue method the LOD, LOQ and expanded measurement uncertainty were described [26]. For the method for the detection of aminoglycosides the linearity, repeatability and reproducibility were measured as well [26]. The quantification was done with one-point standard addition [26]. Because of this, semi-quantitative results were generated.

For the beef cattle FYM samples, two extraction methods were developed. One method (method A) was to identify and semi-quantify 9 aminoglycosides and colistin. This method not suitable for the detection and quantification of neomycin in beef cattle farmyard manure. The other method (method B) was to identify and semi-quantify 59 other antibiotics from different classes (Table 5).

For method A, 2.5 g of each sample was brought into 50 mL polypropylene (PP) tubes (Greiner Bio-One B.V.B.A/S.P.R.L., Vilvoorde, Belgium), and stored at −20 °C until the extraction procedure. After thawing the subsamples, the internal standards ribostamycin and polymyxin B were added. After an equilibration time of 10 min at room temperature, 7.5 mL of extraction buffer containing 10 mM dipotassium hydrogen phosphate (KH_2_PO_4_)–0.4 mM disodium ethylenediaminetetraacetate dihydrate (EDTA-Na_2_)–2% trichloroacetic acid (TCA) (Merck KGaA, Darmstadt, Germany) were added to the samples. The tubes were vortexed, shaken for 10 min at 225–250 rpm and centrifuged for 15 min at 4000× *g*. The supernatant was collected into a clean PP tube and the extraction procedure was repeated on the same pellet, resulting in 15 mL of extract in total. Subsequently, 10 mL HPLC water (high-performance liquid chromatography (HPLC) grade, Milli-Q Gradient purification system, Millipore, Brussels, Belgium) was added and pH was adjusted to 7–8 with a 30% sodiumhydroxide (NaOH, VWR Chemicals BDH^®^, Leuven, Belgium) solution. The extract was purified using solid phase extraction (SPE) columns (Bakerbond spe^TM^ WP-CBX, J.T. Baker, Fisher Scientific, Hampton, New Hampshire, USA). The columns were conditioned with 5 mL methanol (MeOH, LC-MS grade, Biosolve B.V., Valkenswaard, The Netherlands), 5 mL water and 5 mL of 20 mM K_2_HPO_4_ in water. Twenty-five milliliters of extract were loaded and a washing step was performed with 5 mL HPLC water. After drying the columns, the residues were eluted with 2 times 3 mL of elution buffer containing 10% formic acid (FA, Biosolve BV, Valkenswaard, The Netherlands) in methanol. The eluate was evaporated (60 °C, N_2_) and redissolved in 1 mL H_2_O/acetonitrile (ACN, Biosolve BV) (25/75) + 1% FA followed by a filtration step through a 0.22 µm filter (Polyvinylidene fluoride filters, Merck-Millipore, Carrigtwohill, Ireland). Finally 10 µL of the extract was injected into the LC-MS/MS system (Acquity UHPLC, column: Atlantis HILIC silica (2.1 × 150 mm; 3 µm) and analogous pre-column, solvent A: water + 1% FA, solvent B: ACN + 1% FA, Xevo TQ-S mass spectrometer (Waters Corporation)).

For method B, two g of each sample was brought into 50 mL PP tubes and stored at −20 °C until the extraction procedure. After thawing the subsamples, the internal standards were added. Threo-chloramphenicol-d_5_, trimethoprim-d_9_ and sulfadimethoxine ^13^C_6_ were purchased from WITEGA Laboratorien Berlin-Adlershof GmbH (Berlin, Germany). Ceftiofur-d_3_ hydrochloride was purchased from Toronto Research Chemicals (Toronto, Canada). Cincophen, lomefloxacine hydrochloride, clindamycin hydrochloride, methacycline hydrochloride, cefotaxime sodium salt, piperacillin sodium salt and roxithromycin were purchased from Sigma-Aldrich. After an equilibration time of 10 min at room temperature, 4 mL of ACN (MeCN, LC–MS grade, Biosolve BV) was added to the samples, the tubes were vortexed, shaken for 10 min at 225–250 rpm and centrifuged at 4000× *g*. This first supernatant was collected into a clean 50 mL PP tube and the extraction procedure was repeated on the pellet using ACN + 10% FA as extraction buffer. The second supernatant was collected into another clean 50 mL PP tube. After the second supernatant was evaporated (45 °C, under N_2_), the first supernatant was poured onto the evaporated fraction and the whole extract was evaporated (45 °C, under N_2_) again. The evaporated extract was redissolved in 1 mL H_2_O/ACN/MeOH (50/25/25) + 0.05% acetic acid (AA). The extracts were vortexed for 1 min and were sonicated for 5 min in an ultrasonic bath followed by a filtration through a 0.22 µm filter. A 10-fold dilution was made in H_2_O/ACN/MeOH (50/25/25) + 0.05% AA. Finally 10 µL of the extract was injected into the LC-MS/MS system (Acquity UHPLC, column: BEH C_18_ (100 mm × 2.1 mm i.d., 1.7 µm, solvent A: water + 0.05% AA, solvent B: ACN/MeOH (50/50) + 0.05% AA, Xevo TQ-S mass spectrometer (Waters Corporation)).

The quantification for methods A and B differed as follows. The antibiotic residues from method A were quantified using standard addition. Each sample was replicated 4 times. The replicates were spiked with the antibiotics in the following concentrations: 0 ppb, 50 ppb, 200 ppb, 500 ppb, except for tobramycin, which was spiked at 0 ppb, 500 ppb, 750 ppb, 1000 ppb. Although a high variation in the composition of the manure samples was observed, the antibiotic residues from method B were quantified using a matrix matched calibration curve due to practical and financial restrictions. The Limits of Detection (LOD) of the 69 antibiotic residues in beef cattle FYM obtained using method A and B are listed in Table 4 and Table 6. The Limit of Detection (LOD) was calculated as 3 times the standard error of the y-intercept of the regression line divided by the slope. The Limit of Quantification was calculated as 10 times the standard error of the y-intercept of the regression line divided by the slope. For method A, the LOD and LOQ were calculated for each antibiotic in each sample using the calibration curve obtained by standard addition. The LODs were verified with the chromatograms. Afterwards, the mean LOD, the mean LOQ and their coefficients of variation (CV) were calculated, listed in Table 4. Although the mean LOD and mean LOQ were calculated, the LODs and LOQs of the antibiotics in each individual sample were used to interpret the results because high coefficients of variations were observed, probably due to differences in composition of the manure (Table 4). Additionally, for method B, the LOD was also estimated from the chromatograms (Table 6) if it did not approximate the theoretical LOD. The LOQ was calculated if the theoretical LOD was in accordance with the chromatogram check (Table 6). All results obtained by LC-MS/MS were considered semi-quantitative as differences in manure composition can cause different matrix effects and the methods have only been validated to a limited but adequate extent.

## Figures and Tables

**Table 1:** Resistance profiles of *E. coli* isolates from 25 beef cattle farmyard manure samples (number of isolates, *n* = 82) and 9 fattening calf slurry samples (*n* = 41).

Manure Type	Antibiotic Resistance Profile ^1^	Number of *E. coli* (%)
fattening calf slurry	AMP&AZI&CHL&CIP&COL&GEN&NAL&SMX&TET&TMP	3 (7.3%)
	AMP&AZI&FOT&TAZ&CHL&CIP&NAL&SMX&TET&TMP	1 (2.4%)
	AMP&AZI&CHL&CIP&GEN&NAL&SMX&TET&TMP	2 (4.9%)
	AMP&AZI&FOT&CIP&GEN&NAL&SMX&TET&TMP	1 (2.4%)
	AMP&AZI&CHL&CIP&NAL&SMX&TET&TMP	1 (2.4%)
	AMP&CHL&CIP&COL&GEN&NAL&SMX&TET	1 (2.4%)
	AMP&AZI&CHL&NAL&SMX&TET&TMP	1 (2.4%)
	AMP&CHL&CIP&COL&SMX&TET&TMP	1 (2.4%)
	AMP&CHL&CIP&SMX&TET&TMP	2 (4.9%)
	AMP&CHL&SMX&TET&TMP	3 (7.3%)
	AMP&FOT&TAZ&CHL&SMX	2 (4.9%)
	CHL&CIP&NAL&SMX&TMP	1 (2.4%)
	AMP&CHL&SMX&TET	1 (2.4%)
	AMP&CIP&TET&TMP	2 (4.9%)
	AMP&SMX&TET&TMP	6 (14.6%)
	CHL&GEN&SMX&TMP	1 (2.4%)
	AMP&SMX&TMP	2 (4.9%)
	AMP&TET	1 (2.4%)
	SMX&TMP	1 (2.4%)
	TET	3 (7.3%)
	sensitive	5 (12.2%)
beef cattle farmyard manure	AMP&CHL&CIP&GEN&SMX&TET&TMP	1 (1.2%)
	AMP&AZI&CHL&SMX&TET&TMP	1 (1.2%)
	AMP&FOT&TAZ&CHL&SMX&TET	4 (4.9%)
	AMP&CHL&SMX&TET	2 (2.4%)
	AMP&TET	1 (1.2%)
	CIP&NAL	1 (1.2%)
	SMX&TET	1 (1.2%)
	SMX&TMP	2 (2.4%)
	FOT	1 (1.2%)
	TET	5 (6.1%)
	sensitive	63 (76.8%)

^1^ AMP = ampicillin, AZI = azithromycin, CHL = chloramphenicol, CIP = ciprofloxacin, COL = colistin, FOT = cefotaxime, GEN = gentamicin, NAL = nalidixic acid, TAZ = ceftazidime, TET = tetracycline, TMP = trimethoprim, SMX = sulfamethoxazole.

**Table 2:** Number and percentage of *E. coli* isolated from 9 fattening calf slurry samples (number of isolates *n* = 41) and 25 beef cattle farmyard manure samples (*n* = 82) resistant to the antibiotics tested in the EUVSEC panel. *E. coli* isolates were picked up from RAPID’*E. coli* 2 agar plates without antibiotics added.

Antibiotic	Number of Resistant *E. coli* Isolates from Fattening Calf Slurry Samples (%)	Number of Resistant *E. coli* Isolates from Beef Cattle Farmyard Manure Samples (%)
ampicillin	30 (73.2%)	9 (11.0%)
azithromycin	9 (22.0%)	1 (1.2%)
cefotaxime	3 (7.3%)	5 (6.1%)
ceftazidim	2 (4.9%)	4 (4.9%)
chloramphenicol	20 (48.8%)	8 (9.8%)
ciprofloxacin	16 (39.0%)	2 (2.4%)
colistin	5 (12.2%)	0 (0.0%)
gentamicin	8 (19.5%)	1 (1.2%)
meropenem	0 (0.0%)	0 (0.0%)
nalidixic acid	11 (26.8%)	1 (1.2%)
sulfamethoxazole	30 (73.2%)	11 (13.4%)
tetracycline	30 (73.2%)	15 (18.3%)
tigecycline	0 (0.0%)	0 (0.0%)
trimethoprim	28 (68.3%)	4 (4.9%)

**Table 3:** The frequency of detection, mean concentration (µg/kg), minimum concentration (µg/kg), maximum concentration (µg/kg) and median (µg/kg) of antibiotic residues detected in 9 fattening calf slurry samples and 25 beef cattle farmyard manure samples using UHPLC-MS/MS.

Manure Type	Antibiotic Residue	Frequence of Detection (%)	Mean	Min	Max	Median
fattening calf slurry	doxycycline	9 (100.0%)	2776	441	10,881	1873
	oxytetracycline	9 (100.0%)	4078	98	19,522	1810
	ciprofloxacin	9 (100.0%)	48	5	234	26
	enrofloxacin	9 (100.0%)	31	6	161	14
	flumequine	9 (100.0%)	536	3	4494	21
	lincomycin	9 (100.0%)	36	9	141	18
	tilmicosin	8 (88.9%)	162	8	1149	20
	sulfadiazine	8 (88.9%)	10,895	4	84,084	5
	marbofloxacin	7 (77.8%)	16	6	39	7
	tetracycline	6 (66.7%)	45	10	168	24
	sulfadoxine	4 (44.4%)	6	3	10	6
	neomycin	3 (33.3%)	1863	960	3186	1442
	danofloxacin	2 (22.2%)	7	6	8	7
	tylosin	2 (22.2%)	261	17	504	261
	gamithromycin	1 (11.1%)	6	-	-	-
	tylvalosin	1 (11.1%)	44	-	-	-
	sulfamethazine	1 (11.1%)	3	-	-	-
	colistin A	1 (11.1%)	152	-	-	-
	colistin B	1 (11.1%)	88	-	-	-
beef cattle farmyard manure	oxytetracycline	2 (8.0%)	250	28	471	250
	ciprofloxacin	1 (4.0%)	35	-	-	-
	enrofloxacin	1 (4.0%)	80	-	-	-
	paromomycin	1 (4.0%)	50	-	-	-

**Table 4:** Overview of the mean limit of detection (LOD) and the mean Limit of Quantification (LOQ) obtained by standard addition in the individual samples and their coefficients of variation (CV) of the antibiotics (aminoglycosides and colistin) tested in beef cattle farmyard manure using method A. Method A: a double extraction using 10 mM KH_2_PO_4_–0.4 mM EDTA-Na_2_–2% TCA followed by solid phase extraction by cation exchange (Bakerbond WP-CBX) and injected in the UHPLC-MS/MS system with an Atlantis HILIC silica column.

Antibiotic Residue	Mean LOD (µg/kg)	CV of LOD (%)	Mean LOQ (µg/kg)	CV of LOQ (%)
dihydrostreptomycin	64	71	222	68
hygromycin	45	42	149	42
kanamycin	49	56	164	56
paromomycin	88	52	293	52
spectinomycin	66	41	221	41
streptomycin	48	66	160	66
tobramycin	208	58	961	96
apramycin	66	63	219	63
gentamicin	78	58	259	58
colistin A	95	69	318	69
colistin B	58	66	220	63

**Table 5:** Antibiotics analyzed in beef cattle FYM and fattening calf slurry.

Aminoglycosides	β-lactam Antibiotics	Fluoroquinolones	Sulfonamides and Trimethoprim	Macrolides
apramycin	amoxicillin	ciprofloxacin	sulfachloropyridazine	erythromycin A
dihydrostreptomycin	ampicillin	danofloxacin	sulfaclozine	gamithromycin
gentamicin (sum of C1, C1a, C2/C2a)	benzylpenicillin	difloxacin	sulfadiazine	spiramycin
hygromycin B	cloxacillin	enoxacin	sulfadimethoxine	tilmicosin
kanamycin A	dicloxacillin	enrofloxacin	sulfadoxine	tulathromycin
neomycin B	nafcillin		sulfamerazine	tylosin A
paromomycin	oxacillin	norfloxacin	sulfamethazine	tylvasolin
spectinomycin	penicillin V	flumequine	sulfamethoxazole	
streptomycin	cefalexin	marbofloxacin	sulfamethoxypyridazine	**Diaminopyrimidine** **derivatives**
tobramycin	cefalonium	sarafloxacin	sulfapyridine	dapsone
**Polymyxins**	cefapirin (+ metabolite desacetylcefapirin)	**Quinolones**	sulfaquinoxaline	**Tetracyclines**
colistin A		cinoxacin	sulfathiazole	chlortetracycline
colistin B	cefazolin	nalidixic acid	trimethoprim	doxycycline
**Amphenicols**	cefoperazone	oxolinic acid	**Pleuromutilins**	oxytetracycline
chloramphenicol	cefquinome	**Lincosamides**	tiamulin	tetracycline
florfenicol	ceftiofur (+metabolite desfuroylceftiofur cysteine disulfide)	lincomycin	valnemulin	
thiamphenicol		pirlymicin		

**Table 6:** Overview of the limit of detection (LOD) and the mean Limit of Quantification (LOQ) of the antibiotics tested using method B. Method B: an extraction with ACN followed by a second extraction with ACN + 10 % FA. This extract was then injected in a UHPLC-MS/MS system with a BEH C_18_ column.

Antibiotic Residue	LOD (µg/kg)	LOQ (µg/kg)	Antibiotic Residue	LOD (µg/kg)	LOQ (µg/kg)
**β-lactam antibiotics**			**Quinolones**		
amoxicillin	18.2 (75 ^1^)	-	cinoxacin	4.2	14.0
ampicillin	5.4	17.9	nalidixic acid	5.9	19.8
benzylpenicillin	30.8 (50 ^1^)	-	oxolinic acid	3.7	12.4
cefalexin	13.1	29.5	**Fluoroquinolones**		
cefalonium	11.3 (100 ^1^)	-	ciprofloxacin	5.0	16.6
cefapirin	8.2 (20 ^1^)	-	danofloxacin	21.6	72.1
cefazolin	12.1 (50 ^1^)	-	difloxacin	3.3	10.9
cefoperazone	16.3 (100 ^1^)	-	enoxacin	13.4 (5 ^1^)	-
cefquinome	9.4	35.9	enrofloxacin	4.4	14.7
ceftiofur	4.1	13.6	flumequine	2.8	9.2
cloxacillin	7.6	25.3	marbofloxacin	7.2	24.1
desacetylcephapirin	24.0 (50 ^1^)	-	norfloxacin	15.0	50.1
desfuroyceftiofur cysteine disulfide	7.3 (100 ^1^)	-	sarafloxacin	4.1	13.5
dicloxacillin	4.1	13.8	**Sulfonamides and trimethoprim**		
nafcillin	2.9	9.8	sulfapyridine	4.5	14.9
oxacillin	9.9	32.9	sulfachloropyridazine	6.2	18.8
penicillin V	12.4	41.4	sulfaclozine	9.5	31.7
**Tetracyclines**			sulfadiazine	6.6 (10 ^1^)	-
chloortetracycline	10.3	30.5	sulfadimethoxine	5.2	17.3
doxycycline	8.6	28.6	sulfadoxine	5.0	16.6
oxytetracycline	7.1 (15 ^1^)	-	sulfamerazine	5.5 (8 ^1^)	-
tetracycline	5.6	18.6	sulfamethazine	4.8	16.0
**Macrolides**			sulfamethoxazole	3.9	13.1
erythromycin A	5.6	18.5	sulfamethoxypyridazine	5.6	18.8
gamithromycin	5.2	17.4	sulfaquinoxaline	7.5	25.1
spiramycin	2.7	9.2	sulfathiazole	10.4	34.7
tilmicosin	16.6	50.1	trimetoprim	2.4	7.9
tulathromycin	6.3	20.9	**Lincosamides**		
tylosin A	2.9	9.8	lincomycin	2.6	8.7
tylvalosin	3.5	11.8	pirlimycin	1.6	5.2
**Pleuromutilins**			**Amphenicols**		
tiamulin	1.8	6.1	chloramphenicol	1.5 (5 ^1^)	-
valnemulin	2.3	7.7	florfenicol	2.9	9.5
**Diaminopyrimidine derivatives**			thiamphenicol	9.1 (10 ^1^)	-
dapsone	6.9	23.0			

^1^ The LOD was estimated from the chromatograms.

## Data Availability

The data presented in this study are available on request from the corresponding author. The data are not publicly available due to privacy of the institutional data.

## Supplementary Materials

The following are available online at https://www.mdpi.com/article/10.3390/antibiotics10040410/s1, Table S1: Number and percentage of 7 ciprofloxacin-resistant *E. coli* isolates and 6 cefotaxime-resistant *E. coli* isolates from 9 fattening calf slurry samples and 7 ciprofloxacin-resistant *E. coli* isolates and 5 cefotaxime-resistant *E. coli* isolates from 25 beef cattle FYM samples, showing resistance to the antibiotics tested in the EUVSEC panel. *E. coli* isolates were picked up from RAPID’*E. coli* 2 agar plates with ciprofloxacin or cefotaxime added in their ECOFF concentrations for *E. coli* of 0.064 mg/L and 0.025 mg/L respectively, Table S2: Resistance profile of ciprofloxacin-resistant *E. coli* isolates from 9 fattening calf slurry samples (number of isolates, *n* = 7) and 25 beef cattle FYM samples (number of isolates, *n* = 7). *E. coli* isolates were picked up from RAPID’*E. coli* 2 agar plates with ciprofloxacin added in the ECOFF concentration for *E. coli* of 0.064 mg/L, Table S3: Resistance profile in cefotaxime-resistant *E. coli* isolates from 9 fattening calf slurry samples (number of isolates, *n* = 6) and 25 beef cattle FYM samples (number of isolates, *n* = 5). *E. coli* isolates were picked up from RAPID’*E. coli* 2 agar plates with cefotaxime added in the ECOFF concentration for *E. coli* of 0.025 mg/L.
