# Supplementary material for: Presence of Antibiotic Residues and Antibiotic Resistant Bacteria in Cattle Manure Intended for Fertilization of Agricultural Fields: A One Health Perspective
Source: Antibiotics (Basel). 2021 Apr 9;10(4):410. doi: 10.3390/antibiotics10040410 (PMC8069554; doi:10.3390/antibiotics10040410)
Supplement: Supplementary file 1 [file antibiotics-10-00410-s001.pdf]

Supplementary material

Table S1. Number and percentage of 7 ciprofloxacin-resistant *E. coli* isolates and 6 cefotaxime-resistant *E. coli* isolates from 9 fattening calf slurry samples and 7 ciprofloxacin-resistant *E. coli* isolates and 5 cefotaxime-resistant *E. coli* isolates from 25 beef cattle FYM samples, showing resistance to the antibiotics tested in the EUVSEC panel. *E. coli* isolates were picked up from RAPID'*E. coli* 2 agar plates with ciprofloxacin or cefotaxime added in their ECOFF concentrations for *E. coli* of 0.064 mg/L and 0.025 mg/L respectively.

**Manure type**	**Antibiotic**	**Number of ciprofloxacin-resistant *E. coli* resistant to the antibiotic listed (%)**	**Number of cefotaxime-resistant *E. coli* resistant to the antibiotic listed (%)**
fattening calf slurry	ampicillin	7 (100.0%)	6 (100.0%)
	azithromycin	3 (42.9%)	3 (50.0%)
	cefotaxime	2 (28.6%)	6 (100.0%)
	ceftazidim	1 (14.3%)	6 (100.0%)
	chloramphenicol	7 (100.0%)	5 (83.3%)
	ciprofloxacin	7 (100.0%)	3 (50.0%)
	colistin	2 (28.6%)	2 (33.3%)
	gentamicin	6 (85.7%)	2 (33.3%)
	meropenem	0 (0.0%)	0 (0.0%)
	nalidixic acid	6 (85.7%)	3 (50.0%)
	sulfamethoxazole	7 (100.0%)	5 (83.3%)
	tetracycline	6 (85.7%)	4 (66.7%)
	tigecycline	0 (0.0%)	0 (0.0%)
	trimethoprim	6 (85.7%)	4 (66.7%)
beef cattle FYM	ampicillin	6 (85.7%)	5 (100.0%)
	azithromycin	0 (0.0%)	0 (0.0%)
	cefotaxime	2 (28.6%)	5 (100.0%)
	ceftazidim	2 (28.6%)	5 (100.0%)
	chloramphenicol	5 (71.4%)	4 (80.0%)
	ciprofloxacin	7 (100.0%)	2 (40.0%)
	colistin	0 (0.0%)	0 (0.0%)
	gentamicin	4 (57.1%)	1 (20.0%)
	meropenem	0 (0.0%)	0 (0.0%)
	nalidixic acid	5 (71.4%)	1 (20.0%)
	sulfamethoxazole	6 (85.7%)	4 (80.0%)
	tetracycline	6 (85.7%)	4 (80.0%)
	tigecycline	0 (0.0%)	0 (0.0%)
	trimethoprim	6 (85.7%)	3 (60.0%)

Table S2. Resistance profile of ciprofloxacin-resistant *E. coli* isolates from 9 fattening calf slurry samples (number of isolates, n=7) and 25 beef cattle FYM samples (number of isolates, n=7). *E. coli* isolates were picked up from RAPID'*E. coli* 2 agar plates with ciprofloxacin added in the ECOFF concentration for *E. coli* of 0.064 mg/L.

**Manure type**	**Antibiotic Resistance Profile**	**Number of ciprofloxacin-resistant *E. coli* (%)**
fattening calf slurry	AMP&AZI&CHL&CIP&GEN&SMX&TET&TMP	1 (14.3)
	AMP&AZI&CHL&CIP&GEN&NAL&SMX&TET&TMP	2 (28.6)
	AMP&CHL&CIP&COL&GEN&NAL&SMX&TET&TMP	1 (14.3)
	AMP&FOT&CHL&CIP&NAL&SMX&TMP	1 (14.3)
	AMP&CHL&CIP&COL&GEN&NAL&SMX&TET	1 (14.3)
	AMP&FOT&TAZ&CHL&CIP&GEN&NAL&SMX&TET&TMP	1 (14.3)
beef cattle FYM	AMP&FOT&TAZ&CHL&CIP&GEN&NAL&SMX&TET&TMP	2 (28.6)
	AMP&CHL&CIP&GEN&NAL&SMX&TET&TMP	2 (28.6)
	CIP&SMX&TET&TMP	1 (14.3)
	AMP&CHL&CIP&NAL&SMX&TET&TMP	1 (14.3)
	AMP&CIP	1 (14.3)

Table S3. Resistance profile in cefotaxime-resistant *E. coli* isolates from 9 fattening calf slurry samples (number of isolates, n=6) and 25 beef cattle FYM samples (number of isolates, n=5). *E. coli* isolates were picked up from RAPID'*E. coli* 2 agar plates with cefotaxime added in the ECOFF concentration for *E. coli* of 0.025 mg/L.

**Manure type**	**Antibiotic Resistance Profile**	**Number of cefotaxime-resistant *E. coli* (%)**
fattening calf slurry	AMP&FOT&TAZ&CHL&SMX	1 (16.7)
	AMP&AZI&FOT&TAZ&CHL&CIP&COL&NAL&SMX&TET&TMP	1 (16.7)
	AMP&AZI&FOT&TAZ&CHL&CIP&COL&GEN&NAL&SMX&TET&TMP	1 (16.7)
	AMP&FOT&TAZ&CHL&SMX&TET&TMP	1 (16.7)
	AMP&FOT&TAZ	1 (16.7)
	AMP&AZI&FOT&TAZ&CHL&CIP&GEN&NAL&SMX&TET&TMP	1 (16.7)
beef cattle FYM	AMP&FOT&TAZ&CHL&SMX&TET	1 (20.0)
	AMP&FOT&TAZ&CHL&CIP&TET	1 (20.0)
	AMP&FOT&TAZ&CHL&CIP&NAL&SMX&TMP	1 (20.0)
	AMP&FOT&TAZ&CHL&GEN&SMX&TET&TMP	1 (20.0)
	AMP&FOT&TAZ&SMX&TET&TMP	1 (20.0)
